# Multi-omics analysis of an in vitro photoaging model and protective effect of umbilical cord mesenchymal stem cell-conditioned medium

**DOI:** 10.1186/s13287-022-03137-y

**Published:** 2022-09-02

**Authors:** Xiaocang Zou, Dayang Zou, Linhao Li, Renfeng Yu, XianHuang Li, Xingyue Du, JinPeng Guo, KeHui Wang, Wei Liu

**Affiliations:** 1 Academy of Military Medical Sciences, Academy of Military Sciences, Beijing, 100850 China; 2 Center for Disease Control and Prevention of PLA, 20 Dongdajie Street, Fengtai District, Beijing, 100071 China; 3 The People’s Liberation Army 965 Hospital, JiLin, 132000 China

**Keywords:** Photoaging, SASP, Multi-omics analysis, Umbilical cord stem cells, Conditioned medium

## Abstract

**Background:**

Skin ageing caused by long-term ultraviolet (UV) irradiation is a complex biological process that involves multiple signalling pathways. Stem cell-conditioned media is believed to have anti-ageing effects on the skin. The purpose of this study was to explore the biological effects of UVB irradiation and anti-photoaging effects of human umbilical cord mesenchymal stem cell-conditioned medium (hUC-MSC-CM) on HaCaT cells using multi-omics analysis with a novel cellular photoaging model.

**Methods:**

A cellular model of photoaging was constructed by irradiating serum-starved HaCaT cells with 20 mJ/cm^2^ UVB. Transcriptomics and proteomics analyses were used to explore the biological effects of UVB irradiation on photoaged HaCaT cells. Changes in cell proliferation, apoptosis, and migration, the cell cycle, and expression of senescence genes and proteins were measured to assess the protective effects of hUC-MSC-CM in the cellular photoaging model.

**Results:**

The results of the multi-omics analysis revealed that UVB irradiation affected various biological functions of cells, including cell proliferation and the cell cycle, and induced a senescence-associated secretory phenotype. hUC-MSC-CM treatment reduced cell apoptosis, inhibited G1 phase arrest in the cell cycle, reduced the production of reactive oxygen species, and promoted cell motility. The qRT-PCR results indicated that MYC, IL-8, FGF-1, and EREG were key genes involved in the anti-photoaging effects of hUC-MSC-CM. The western blotting results demonstrated that C-FOS, C-JUN, TGFβ, p53, FGF-1, and cyclin A2 were key proteins involved in the anti-photoaging effects of hUC-MSC-CM.

**Conclusion:**

Serum-starved HaCaT cells irradiated with 20 mJ/cm^2^ UVB were used to generate an innovative cellular photoaging model, and hUC-MSC-CM demonstrates potential as an anti-photoaging treatment for skin.

**Supplementary Information:**

The online version contains supplementary material available at 10.1186/s13287-022-03137-y.

## Background

Skin ageing results from a combination of internal and external factors that lead to the decline of skin structure integrity and destruction of normal physiological function. Among them, ultraviolet (UV) light is the most important external factor that causes exogenous skin ageing, also known as photoaging [1]. The damage caused by UV rays varies depending on the wavelength [2, 3], with 800–1000 times more skin damage caused by UVB radiation than by the same dose of UVA radiation [4]. UVB radiation can cause DNA damage, resulting in skin erythema and skin cancer.

Building cellular ageing models is a common method used to study photoaging. Diploid cells undergo accelerated senescence independent of telomere shortening, termed premature senescence, after exposure to certain insults [5]. UVB exposure causes premature skin ageing [6]. Although most cellular photoaging studies use 10% foetal bovine serum (FBS) as a growth supplement [7, 8], the composition of FBS is complex and its effect on the UVB response of cells remains unknown. The study had shown that autologous topical serum based on growth factor-rich plasma technology has notably anti-photoaging properties [9]. Various serum growth factors, such as transforming growth factor β1 (TGF β1) and epidermal growth factor (EGF), can promote the transcription and synthesis of proteins involved in skin regeneration [10–12]. Therefore, this study aimed to construct a cellular model of photoaging without serum.

Exploring the biological processes underlying photoaging is a research hot spot [13–15]. However, using multi-omics analysis in photoaging studies is rare and its analysis can help elucidate the complex biological changes at different levels during the photoaging process [16]. Senescent cells not only exhibit growth cycle arrest, but they also secrete many cytokines through autocrine and paracrine pathways that affect the microenvironment of neighbouring cells and tissues. The characteristics exhibited by senescent cells contribute to the senescence-associated secretory phenotype (SASP) [17]. Typical SASP factors include tumour necrosis factor-α (TNF-α), interleukin-6 (IL-6), interleukin-1 (IL-1), interleukin-8 (IL-8), matrix metalloproteinases (MMPs), granulocyte colony-stimulating factor (GCSF), and plasminogen activator inhibitor-1. These factors stimulate the activation of the immune system. Furthermore, in addition to inhibiting tumorigenesis and promoting the repair of damaged tissue, SASP factors can also directly or indirectly lead to chronic inflammatory responses in the body. Chronic inflammation is a key mechanism for ageing-related diseases [18]. Many inducing factors can lead to premature accumulation of senescent cells, leading to SASP, among which UV light effectively induces SASP in skin cells [19].

Stem cells are an area of interest in anti-photoaging research because of their unlimited proliferation potential and multi-directional differentiation ability. Stem cells can reduce oxidative stress, inhibit apoptosis and ageing, promote the synthesis of extracellular matrix (ECM) and skin regeneration, regulate inflammatory processes, and have anti-wrinkle and whitening effects [1]. Several studies have confirmed that stem cells can resist photoaging and restore the barrier function of ageing skin [20–22]. However, the use of stem cells has certain limitations, including maintaining biological activity, quantification of biologically active substances, and logistics and distribution [23]. However, stem cells secrete a variety of bioactive molecules through paracrine mechanisms that affect the surrounding microenvironment, suggesting that stem cell-conditioned medium may be an effective replacement for stem cell therapy. Stem cell-conditioned medium contains many growth factors, such as ILs, insulin, TNF-α, vascular endothelial growth factor, hepatocyte growth factor, and GCSF, which can protect tissue by reducing apoptosis and inhibiting oxidative stress [24]. Furthermore, exosomes in stem cell-conditioned medium have also been shown to exert anti-photoaging effects [25]. Compared to other types of stem cells, human umbilical cord mesenchymal stem cells (hUC-MSCs) can be collected by non-invasive means, have lower immunogenicity, and exhibit faster proliferation, making them the first choice for regenerative medicine [26]. Liu et al. applied UC-MSC serum-free medium (UC-MSC SFM) to a photoaging model constructed using human dermal fibroblast (HDF) cells, reporting that UC-MSC SFM promoted fibroblast proliferation and increased superoxide dismutase and glutathione peroxidase (GPX) activities in a dose-dependent manner [27].

The International Cell Senescence Association has identified cell cycle arrest, SASP, macromolecular damage, chromatin landscape, deregulated metabolic profile, transcriptional signatures, miRNAs, non-coding RNAs, and immune-regulation and anti-apoptotic proteins as key features for evaluating cellular senescence [28]. Therefore, the objective of this study was to evaluate changes in some of these ageing biomarkers to determine whether hUC-MSC-CM exerts an anti-ageing effect on UVB-induced, serum-starved HaCaT cells.

## Methods

### Cell culture and construction of a photoaging model

HaCaT cells and hUC-MSCs were purchased from Procell (Wuhan, Hubei, China). Complete cell growth medium (CGM) comprised Dulbecco’s Modified Eagle Medium (DMEM) (Gibco, Grand Island, NY, USA) containing 10% FBS (Gibco) and 1% penicillin–streptomycin (Gibco). Cells were cultured in 60 mm cell culture dishes at 37 °C in a 5% CO_2_ incubator. All cells were verified to be mycoplasma contamination-free using the Mycoplasma Detection Kit (Solarbio, Beijing, China).

When HaCaT cells reached approximately 60% confluence, the complete growth medium was replaced with serum-free cell culture medium and cells were starved for 24 h. Subsequently, the starved cells were divided into control (Ctrl) and UVB-treated (UVB) groups. The UVB group was irradiated for 40 s at a distance of 20 cm using an SH2B type UVB lamp (SIGMA, Shanghai, China) at an intensity of 500 μW/cm^2^ and an irradiation dose of 20 mJ/cm^2^. During UVB irradiation, cells were covered with a small amount of phosphate-buffered saline (PBS) to prevent direct exposure. After irradiation, CGM was added to both groups and cells were cultured for an additional 24 h. The Ctrl group was treated with natural light, and all other operational steps were the same as those described for the UVB group.

### RNA isolation and library construction

After 24 h, the medium was discarded, and cells were washed twice with PBS. TRIzol reagent (BBI, Shanghai, China) (1 mL) was added to each cell culture dish, and cells were rested for 10 min. The liquid was transferred to an RNase-free EP tube, to which 0.2 mL of chloroform was added. The tube was vigorously shaken for 15 s and then allowed to rest for 3 min. The mixture was centrifuged at 12,000 × *g* for 10 min at 4 °C, and the upper aqueous phase was transferred to a new EP tube, to which an equal volume of isopropanol was added. After 20 min, the tube was centrifuged at 12,000 × *g* for 10 min at 4 °C, and the supernatant was discarded. The RNA pellet was washed twice with 1 mL of 75% ethanol and centrifuged at 12,000 × *g* for 3 min at 4 °C. The RNA pellet was air-dried for approximately 5 min, and 50 μL DEPC-treated water was added to dissolve the RNA. RNA concentration was determined using a QUBIT 3.0 fluorometer (Thermo Fisher Scientific, Waltham, MA, USA). Library construction and sequencing were performed by the BGI Group (Shenzhen, China). Briefly, total RNA was isolated, and mRNA was enriched using oligo (dT) primers coupled to magnetic beads. Double-stranded cDNA was synthesised by reverse transcription using N_6_ primers. The end of the synthesised double-stranded cDNA was blunted, the 5′ end was phosphorylated, the 3′ end was bonded to ‘A’, and a linker with a protruding ‘T’ was connected. The ligated products were amplified by PCR using specific primers. The PCR product was heat-denatured to single-stranded cDNA, which was circularised using splint oligo and DNA ligase. Finally, the cDNA library was sequenced using the MGISEQ-2000 sequencing platform (MGI Tech Co., Ltd., Shenzhen, China).

### Bioinformatics analysis

Clean reads were obtained by filtering the sequencing data using SOAPnuke v1.5.2 (https://github.com/BGI-flexlab/SOAPnuke). Clean reads were mapped to the reference genome using HISAT2 v2.0.4 (http://www.ccb.jhu.edu/software/hisat/index.shtml). Ericscript v0.5.5 (http://ericscript.sourceforge.net/) and rMATS v3.2.5 (http://rnaseq-mats.sourceforge.net) were used to analyse fusion genes and differential splicing genes (DSGs). Clean reads were aligned with known and new coding transcripts in the biological gene database established by the Beijing Institute of Genomics (Shenzhen, China) using HISAT2 v2.0.4. Gene expression levels were calculated using RSEM v1.2.12 (https://github.com/deweylab/RSEM). Heatmaps were drawn using the pheatmap package v1.0.8 in R (https://cran.r-project.org/web/packages/pheatmap/index.

html). Differential expression analysis was performed using DESeq2 v1.4.5 (http://www.bioconductor.org/packages/release/bioc/html/ DESeq2.html). The *Q* values were corrected using the Bonferroni method (*Q* ≤ 0.05).

### Protein extraction, peptide labelling, and identification

HaCaT cells were lysed on ice by sonication for 5 min in a cocktail containing EDTA, followed by centrifugation at 25,000 × *g* for 15 min at 4 °C. The supernatant was removed, the proteins were reduced and alkylated using 10 mM DTT and 55 mM iodoacetamide, and the mixture was centrifuged at 25,000 × *g* for 15 min at 4 °C. Protein concentration was quantified using the Bradford method. Subsequently, proteins were trypsinised at 37 °C for 4 h, desalted, and freeze dried. The samples were reconstituted with 2 mM TEAB and labelled with the corresponding iTRAQ reagents at 37 °C for 2 h. The labelled peptides were separated on a Gemini C18 column (Phenomenex, Torrance, CA, USA) using the Shimadzu LC-20AD liquid chromatography system (Shimadzu, Kyoto, Japan), and the 20 resulting fractions were lyophilised. The peptides were further separated using the UltiMate 3000 UHPLC system (Thermo Fisher Scientific). The separated ends were detected using a Q Exactive HF-X Hybrid Quadrupole-Orbitrap mass spectrometer (Thermo Fisher Scientific, San Jose, CA, USA) operated in data acquisition mode. The raw MS/MS data were converted into MGF format and aligned with the NCBI database using Mascot v2.3.02 identification software.

### Protein analysis

Proteins were quantified using the IQuant automation software [29]. Initially, reads were filtered with a 1% false discovery rate (FDR) at the spectrum/peptide level (PSM-level FDR ≤ 0.01). To control the false positive rate at the protein level, the reads were again filtered at the protein level with 1% FDR based on the selected protein FDR strategy [30]. Subsequently, differentially expressed proteins were analysed using Gene Ontology (GO) aggregation analysis, Kyoto Encyclopedia of Genes and Genomes (KEGG) pathway analysis, and functional annotation. Data were analysed by one-way analysis of variance (ANOVA) (fold change > 1.2, *p* < 0.05).

### Collection of hUC-MSC-CM

Between third and fifth generation, hUC-MSCs were used for experiments. Cells were seeded in T75 culture flasks. To control for the single experimental variable, hUC-MSCs were cultured in a high-glucose medium containing 10% FBS, consistent with the culture medium used for HaCaTs. When the hUC-MSCs reached 70% confluence, the culture medium was collected and centrifuged at 1000 rpm for 5 min to produce hUC-MSC-CM.

### Experimental grouping

HaCaT cells were divided into three groups: Ctrl (UVB-, CGM), UVB (UVB+, CGM), and hUC-MSC-CM (UVB+, hUC-MSC-CM). The groups received the same lighting treatments described for the construction of the cellular photoaging model. CGM was added to the Ctrl and UVB groups after lighting treatment, and cells were cultured for an additional 24 h. The same volume of hUC-MSC-CM as CGM was added to the hUC-MSC-CM group after UVB irradiation and cells were cultured for 24 h.

### Proliferation assay

Normal and photoaged HaCaT cells were seeded in 96-well plates at a density of 1 × 10^4^ cells/well. CGM was added to HaCaT cells in the Ctrl and UVB groups, whereas hUC-MSC-CM was added to the photoaged cells in the hUC-MSC-CM group. Each group contained three replicate wells. Cells were cultured for 24, 48, and 72 h at 37 °C in a 5% CO_2_ incubator. CCK-8 (Solarbio, Beijing, China) (10 μL) was added to each well, and cells were incubated at 37 °C for 30 min. Absorbance was measured at 450 nm using a microplate reader.

### Apoptosis detection

The cells in each group were digested with trypsin, which was terminated by adding the original medium. Next, cells were centrifuged at 1000 rpm for 5 min and washed twice with PBS. Annexin V-FITC binding buffer (195 μL) was added to resuspend the cells, followed by 5 μL Annexin V-FITC (Abcam, Cambridge, UK). Cells were incubated at 37 °C for 10 min in the dark, followed by centrifugation at 200 × *g* for 5 min, and the supernatant was discarded. Cells were resuspended in 190 μL Annexin V-FITC binding solution, and then, 10 μL propidium iodide (PI) was added to the suspension, which was thoroughly mixed. Subsequently, the cells were incubated in the dark for 10 min, and cell apoptosis was detected using a FACSCalibur flow cytometer (BD Biosciences, San Jose, CA, USA).

### Cell cycle detection

Cells in each group were collected, washed twice with PBS, resuspended in 1 mL of 70% cold ethanol, and fixed overnight at 4 °C. The cells were centrifuged, and the supernatant was discarded. RNaseA (final concentration 20 μg/mL) was added to cells, followed by incubation at 37 °C for 30 min. Cells were washed with PBS, centrifuged, and stained with PI in the dark for 30 min. The cell cycle stage was detected using a FACSCalibur flow cytometer (BD Biosciences).

### Detection of reactive oxygen species

The fluorescent probe CellROX^™^ Deep Red Reagent (Invitrogen, Carlsbad, CA, USA) was used to detect the reactive oxygen species (ROS) content of cells in each group. Cells were stained with acridine orange dye and incubated for 30 min. The images were captured using an IN Cell Analyzer 2000 (GE Healthcare, Marlborough, MA, USA) to observe and calculate the fluorescence value by high-content screening.

### Cell migration assay

Cells were seeded in 96-well plates at a density of 4 × 10^4^ cells/well. When cell confluence reached 100%, cells in the Ctrl, UVB, and hUC-MSC-CM groups underwent lighting treatments as previously described. Subsequently, a sterile scratcher was used to scratch the surface of each well. Cell migration through the scratch was recorded at 0, 15, and 30 h using the Incucyte^®^ cell imaging system (Essen BioScience Ltd., Ann Arbor, MI, USA).

### qRT-PCR

Total RNA was extracted using TRIzol. First-strand cDNA was synthesised using the PrimeScript^™^ RT Kit (Takara Bio, Kusatsu, Japan). PCR reactions were performed using the CFX Connect Real-Time PCR Detection System (Bio-Rad, Hercules, CA, USA) using TB Green^®^ Premix Ex Taq^™^ (Tli RNaseH Plus) (Takara Bio). The relative expression of mRNA was calculated using the 2^−ΔΔCT^ method. GAPDH was the internal reference for gene expression. Each sample was analysed in triplicate. The primers used for this experiment are listed in Additional file 1: Table S1.

### Western blot analysis

Cells were collected and lysed with protease and phosphatase inhibitors. The protein concentration of the samples was determined using a BCA Protein Analysis Kit (Sangon Biotech, Shanghai, China). The proteins in the sample (20 μL) were separated by electrophoresis on 12% SDS-PAGE gel and proteins were transferred to a PVDF membrane using a semi-dry transfer system (Trans-Blot SD Semi-Dry Electrophoretic Transfer Cell; Bio-Rad). The membrane was blocked with 5% non-fat dry milk for 2 h and then incubated overnight at 4 °C with rabbit anti-human primary antibody (Abcam, Cambridge, UK), GAPDH (1:2000), FGF1 (1:2000), p53 (1:1000), C-FOS (1:2000), C-JUN (1:2000), TGFβ (1:2000), and cyclin A2 (1:2000) antibodies (Abcam, Cambridge, UK). After washing with TBST three times, the membrane was incubated with HRP-labelled anti-rabbit secondary antibody (Abcam) for 2 h at 37 °C. Finally, ECL luminescence was used to visualise the protein bands. GAPDH was the internal reference.

### Statistical analysis

Statistical analysis was performed using IBM SPSS Statistics v20.0 software (IBM Corp., Armonk, NY, USA). GraphPad Prism 5 (GraphPad Software, La Jolla, CA, USA) and Origin 2017 (OriginLab, Northampton, MA, USA) were used to visualise the data with graphs. The results are expressed as the mean ± standard deviation. For comparisons between groups, two-tailed paired Student’s *t* test was used when variances were similar (tested with *F*-test), and two-tailed unpaired Student’s *t* test with Welch’s correction was used when variances were different.

## Results

### Transcriptomics

**Fig. 1:**
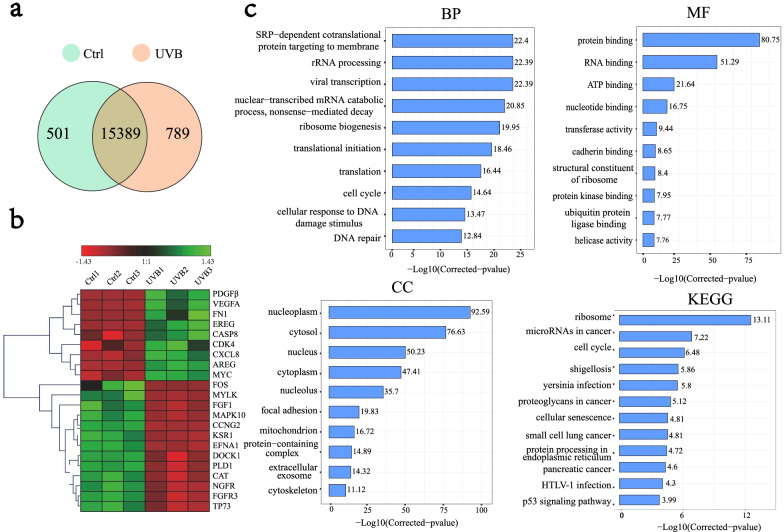
Functional annotation of differential genes before and after UVB illumination. **a** VENN map of gene changes before and after light exposure to the transcriptome. A circle represents a gene set, overlapping indicates common genes, non-overlapping indicates unique genes, and numbers represent the number of genes in the corresponding region. **b** Heatmap of partial differential gene clustering. The horizontal represents different groups, the vertical represents the gene, and the colour represents gene expression. (Red represents a higher gene expression level, and green represents lower gene expression level.) **c** Enrichment of differential genes, KEGG and GO pathways. The abscissa represents − log10 (corrected *p* value): the larger the value, the higher its significance. The ordinate displays the name of the GO or KEGG pathway

The enriched functions of differentially expressed mRNAs were analysed to explore the effect of UVB exposure on the transcriptome of HaCaT cells using the phyper function in R (|log2FC| > 0, *p* < 0.05). 15,389 common genes were identified before and after UVB exposure (Fig. 1a). Differences in the expression of partial key genes in the transcriptome are shown in Fig. 1b. The GO terms included biological processes (BP), molecular functions (MF), and cellular components (CC) (Fig. 1c). The enriched BP terms were mainly SRP-dependent co-translational protein targeting to the membrane (22.4), rRNA processing (22.39), viral transcription (22.39), translation (16.44), cell cycle (14.64), cellular response to DNA damage stimulus (13.47), and DNA repair (12.84). Differential genes were enriched in MF terms, mainly focused on binding and enzyme activities. The enriched CC terms mainly included nucleus (92.59), cytosol (76.63), nucleus (50.23), cytoplasm (47.41), nucleus (35.7), and focal adhesion (19.83). The annotations of the KEGG pathway score mainly involved ribosomes (13.11), microRNAs in cancer (7.22), the cell cycle (6.48), cellular senescence (4.81), protein processing in the endoplasmic reticulum (4.72), and the P53 signalling pathway (3.99).

The biological process of SASP, demonstrated in Additional file 2: Fig. S1, is closely related to cellular senescence. DNA damage activates p53, which can lead to an increase in p21, which is a checkpoint in the G1 phase of the cell cycle and is involved in the regulation of the cell cycle [31]. p21 can also regulate CDK2 to inhibit inactivation of Cdh1 phosphorylation, target EHMT1, and EHMT2 histone methyltransferases, inhibit the silencing of IL-6 and IL-8 promoters, and increase their transcription [32]. RAS signalling activates the CEBPB transcription factor and regulates the transcription of IL-6 and IL-8 [33]. IL1A is a minor component of SASP, and its transcription is regulated by the AP-1 (C-FOS, C-JUN) complex [34]. SASP-related factors can be classified as receptor-requiring, direct-acting, and regulatory SASP factors. Receptor-requiring SASP factors include interleukins (IL-1, IL-6, IL-8), chemokines (CXCL-1, CCL-2), and growth factors (FGF, TGF). Direct-acting SASP factors include MMPs and serine proteases (uPA, tPA). SASP regulatory factors include plasminogen activator inhibitors, tissue metalloproteinase inhibitors, and insulin-like growth factor-binding proteins [35]. Furthermore, UV light activates nuclear factor kappa B (NF-κB), which can induce increased transcription of pro-inflammatory factors IL-1 and TNF-α [36]. MMP-9 is also a classic molecular marker of SASP [37]. Catalase (CAT) and GPX are important antioxidants with anti-photoaging abilities [7]. Table 1 lists the transcriptome data for the key genes involved in the SASP process. Most of the data aligned with trends for these genes during the SASP process, indicating that HaCaT cells could express the SASP phenotype after being starved of serum and irradiated with 20 mJ/cm^2^ UVB. The expression of p21 was greatly decreased in the transcriptome, which may be related to the increase in MYC and JUN expression. Various mechanisms regulate p21 levels in cells, including transcriptional regulation, epigenetic silencing, mRNA stability, as well as ubiquitin-dependent and ubiquitin-independent protein degradation [38]. MYC recruits Dnmt3, a DNA methyltransferase corepressor, to the p21 promoter, repressing p21 transcription [39]. C-JUN can inhibit the transcription of p21 by acting on the Sp1-3 site [40].

**Table 1:** Expression of genes closely related to SASP in the transcriptome

Gene ID	Gene symbol	Gene name	Chromosome	Log2 FC
7157	TP53	Tumour protein p53	17p13.1	0.23
1026	CDKN1A (P21)	Cyclin dependent kinase inhibitor 1A	6p21.2	− 1.04
1017	CDK2	Cyclin dependent kinase 2	12q13.2	0.32
79,813	EHMT1	Euchromatic histone lysine methyltransferase 1	9q34.3	0.29
10,919	EHMT2	Euchromatic histone lysine methyltransferase 2	6p21.33	0.162
3569	IL-6	Interleukin-6	7p15.3	0.78
3576	CXCL8 (IL-8)	C-X-C motif chemokine ligand 8	4q13.3	2.23
1051	CEPBP	CCAAT enhancer binding protein beta	20q13.13	0.29
3552	IL1A	Interleukin 1 alpha	2q14.1	2.25
3725	C-JUN	Jun proto-oncogene	1p32.1	0.93
4318	MMP-9	Matrix metallopeptidase 9	20q13.12	2.37
7124	TNF	Tumour necrosis factor	6p21.33	2.04
5054	SERPINE1 (PAI)	Serpin family E member 1	7q22.1	1.19
4001	LMNB1	Lamin B1	5q23.2	− 0.37
847	CAT	Catalase	11p13	− 0.97
2877	GPX2	Glutathione peroxidase 2	14q23.3	− 0.74
4609	MYC	MYC proto-oncogene	8q24.21	1.01

### Proteomics

**Fig. 2:**
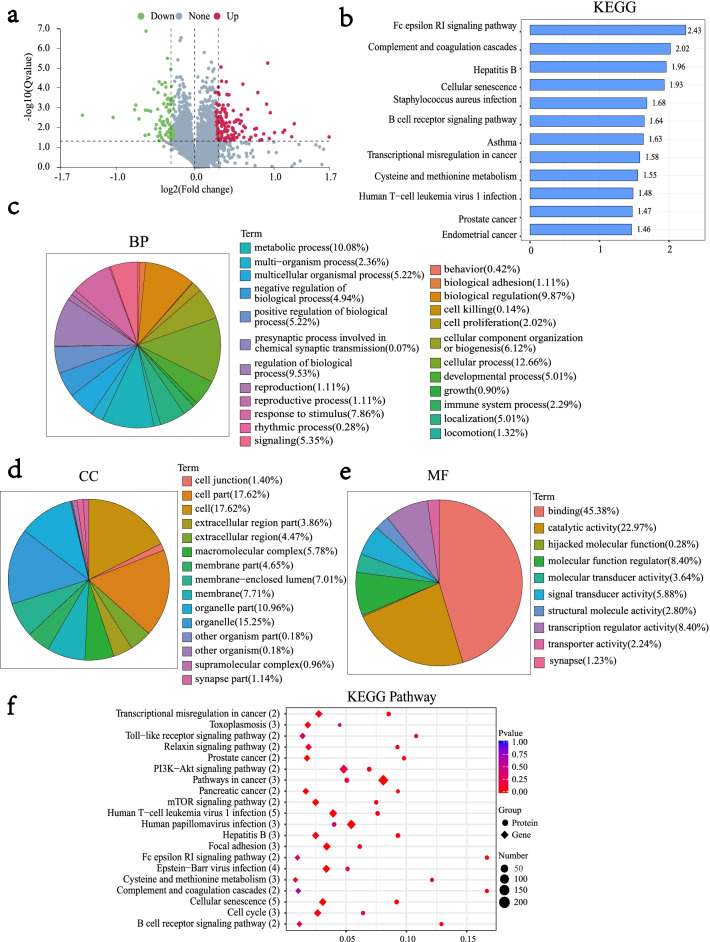
Functional annotation of differential proteins and their association with the transcriptome after UVB irradiation. **a** Significant distribution of differential proteins. The *X*-axis represents the fold difference in protein expression (take log2), the *Y*-axis represents the corresponding − log10 (*Q*-value), the red dots indicate proteins with increased expression and the green dots indicate proteins with decreased expression. The grey point indicates no significant change, where fold change > 1.2 *Q*-value < 0.05. **b** Differential protein KEGG pathway enrichment analysis. The *X*-axis represents − log10 (corrected *p* value): the larger the value, the greater its significance. The ordinate displays the KEGG pathway name. **c**, **d**, **e** Differential protein GO pathway enrichment analysis. **f** Joint analysis of differential proteins and transcriptomes. The *Y*-axis represents each pathway, the number of proteins associated with the entry is shown in parentheses, the *X*-axis indicates the enrichment factor; the bubbles of different shapes represent the data of different omics, and the circles represent the enrichment results of the proteome. Diamonds represent the enrichment results of the transcriptome. The size of the bubble represents the number of differential proteins or genes enriched in the term; the colour represents the significantly enriched *p* value. The larger the enrichment factor, the greater the proportion of differential proteins or differential genes in this type of function; the smaller the *p* value, the more significant the enrichment in this type of function

The differential protein distribution is shown in Fig. 2a. Functional annotation of differential proteins was performed by GO and KEGG enrichment analyses (|log2FC| > 1.2, *p* < 0.05). Differential proteins were enriched in KEGG pathways associated with the Fc epsilon RI signalling pathway and cellular senescence (Fig. 2b) and BP terms such as cellular process (12.66), metabolic process (10.08), biological regulation (9.87), and regulation of biological process (9.53) (Fig. 2c). The enriched CC terms mainly included the cell part (17.62), cell (17.62), and organelle (15.25) (Fig. 2d). Furthermore, the enriched MF terms were binding (45.38), catalytic activity (22.97), molecular function regular (8.40), and transcription regulator activity (8.40) (Fig. 2e).

The top 20 KEGG pathways simultaneously enriched in both the transcriptome and proteome are shown in Fig. 2f. Excluding pathways irrelevant to our research objective, the transcriptional misregulation in the cancer pathway is mainly related to the occurrence and development of cancer. UVB can cause direct damage to the skin, resulting in biological changes in DNA, RNA, and proteins that lead to cell senescence and apoptosis. Furthermore, DNA damage can also destroy the apoptotic ability of cells and increase the risk of tumorigenesis [41]. Focal adhesion is an important pathway that affects cell migration [42]. Cellular senescence and cell cycle signalling pathways are key pathways closely related to cellular ageing. Moreover, the number of associated proteins enriched in cellular senescence signalling pathway is five, which is also the largest.

### Effect of hUC-MSC-CM on proliferation, apoptosis, and cell cycle of UVB-irradiated HaCaT cells

**Fig. 3:**
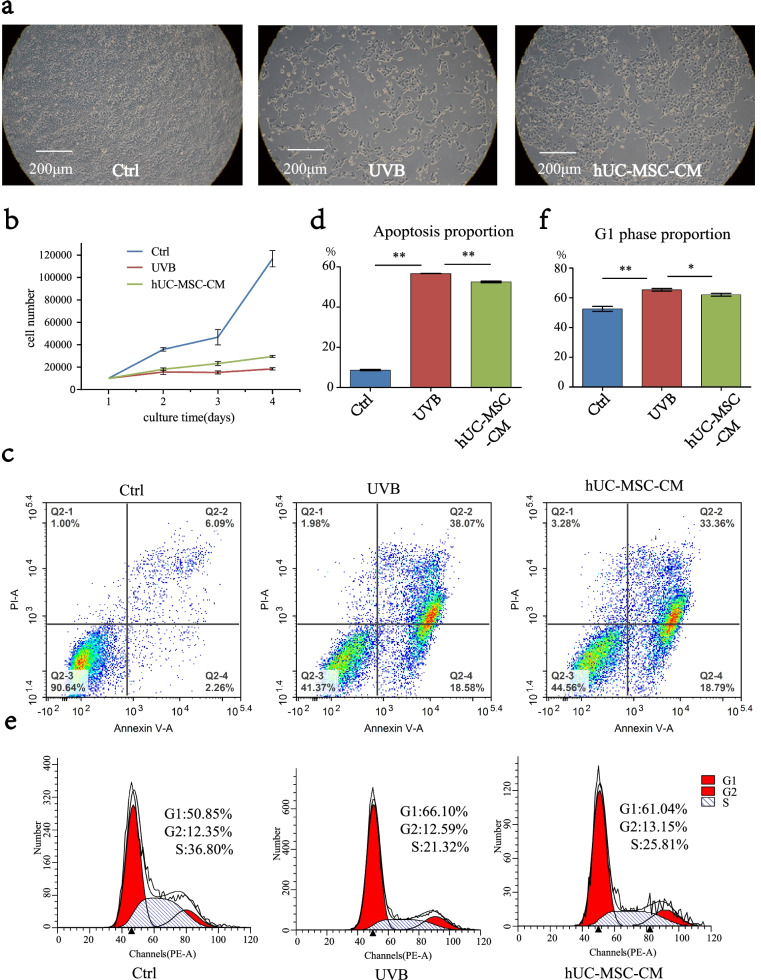
Effects of hUC-MSC-CM on proliferation, cell cycle, and apoptosis of UVB-irradiated HaCaT cells. **a** Cell morphology, viewed as 10 × 10 cell microscope images. **b** Cell proliferation. The *X*-axis represents the cell culture time, the vertical axis represents the total number of cells, and different groups are represented by different line segments. Statistical results are expressed as mean ± standard deviation (*n* = 3). **c**, **d** Changes in cell apoptosis. **e**, **f** Changes in the cell cycle. **, *p* < 0.01; *, *p* < 0.05; *n* = 3

UVB radiation can cause DNA damage, cellular senescence, and even cancerisation of skin cells [43]. Apoptosis induced by UVB irradiation is believed to be a protective response that maintains skin thickness and eliminating mutated precancerous cells [44]. Cellular senescence is accompanied by a decrease in the ability of cells to proliferate and an arrest in the G1 phase of the cell cycle [45]. Meanwhile, cell cycle arrest is considered a key feature of cellular senescence [5]. Epigenetically, exposure to light can cause an increase in cell apoptosis and swelling. In the current study, photoaged HaCaT cells treated with hUC-MSC-CM exhibited a decrease in the proportion of apoptosis and partial recovery of cell morphology (Fig. 3a). UVB-induced changes in cell proliferative capacity were detected using the CCK-8 assay (Fig. 3b), whereas those in apoptosis (Fig. 3c, d) and cell cycle (Fig. 3e, f) were detected by flow cytometry. After HaCaT cells were irradiated with 20 mJ/cm^2^ UVB, apoptosis increased by 48.3% and the proportion of cells in the G1 phase increased by 15.25% compared to the control group. These results demonstrated that UVB exposure led to increased apoptosis and G1 phase arrest in HaCaT cells. In the hUC-MSC-CM group, the proportion of apoptosis decreased by approximately 4.5% compared to the UVB group, whereas the proportion of cells in the G1 phase decreased by approximately 5.06%. Figure 3d, f provides statistics for the triplicate results of apoptosis and cell cycle. These results indicated that hUC-MSC-CM could inhibit UVB-induced apoptosis of HaCaT cells, while also alleviating G1 phase arrest. Furthermore, UVB exposure led to decreased cell proliferation, whereas treatment with hUC-MSC-CM inhibited this trend.

### Effect of hUC-MSC-CM on ROS generation and cell migration UVB-irradiated HaCaT cells

**Fig. 4:**
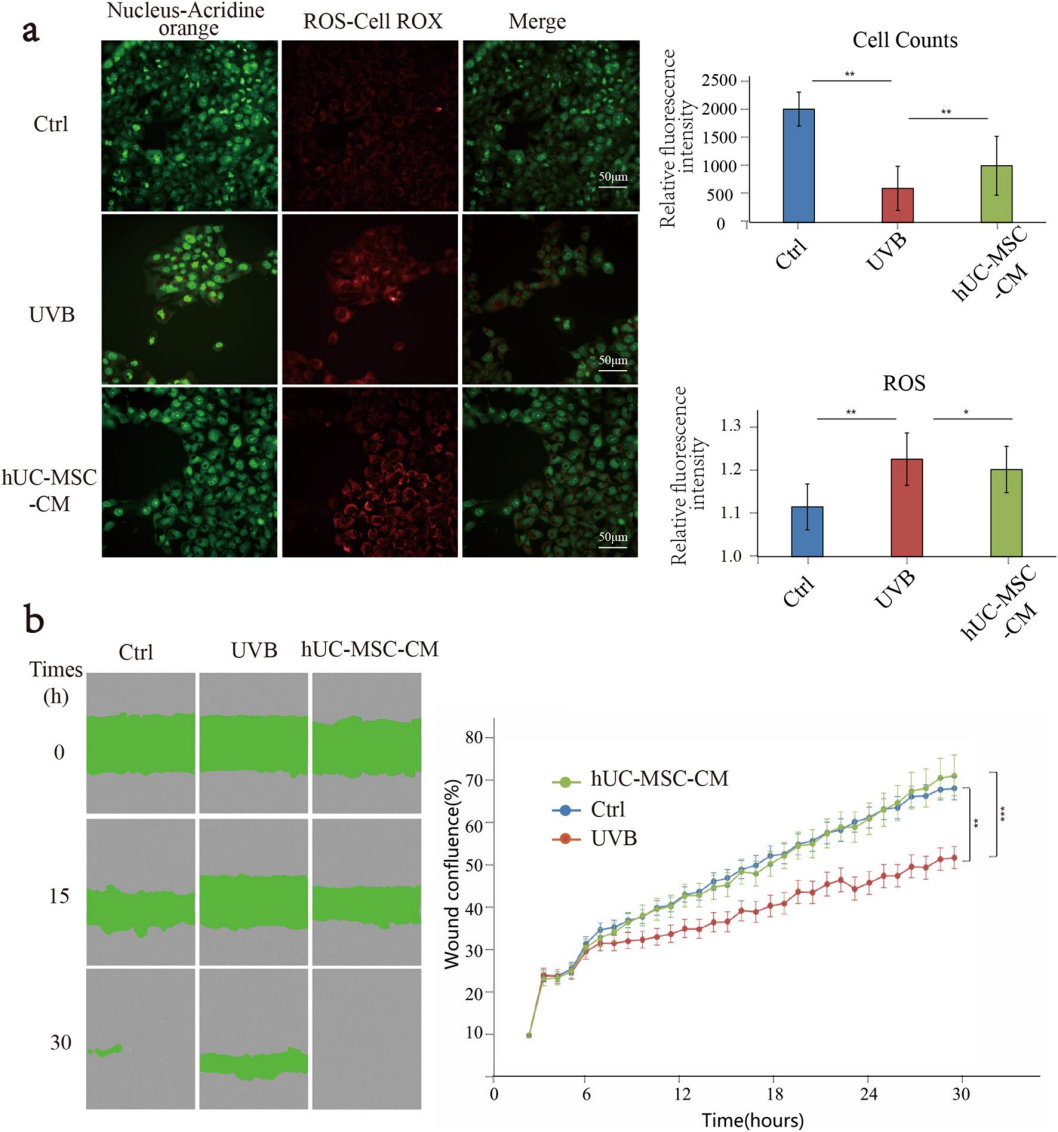
Effects of hUC-MSC-CM on ROS generation and cell migration in UVB-irradiated HaCaT cells. **a** ROS production. Images on the left display results under fluorescence microscopy. The graphs on the right represent the fluorescence result statistics. The horizontal axis of the graph on the right represents different groups, whereas the vertical axis represents the relative fluorescence intensity. The above statistical graph results from the cell count, and the following are the statistical results of ROS. **, *p* < 0.01; *, *p* < 0.05. **b** Cell scratch assay. The left side displays images of cell scratches under the microscope, the horizontal represents different groups, and the vertical represents different times (0, 15, 30 h). The grey part indicates overgrown cells, and the green part represents the scratch condition. The panel on the right displays the statistical results of the scratch convergence. The extent of scratch closure was expressed as the wound confluence. Statistical results were expressed as the mean ± standard deviation (*n* = 3)

ROS is an important factor involved in cell damage caused by light. The ability of HaCaT cells to generate ROS was enhanced after UVB irradiation, whereas the ROS content decreased after the hUC-MSC-CM intervention (Fig. 4a). These results suggested that hUC-MSC-CM has an anti-ROS generation effect. The effect of hUC-MSC-CM on HaCaT cell motility after UVB irradiation is shown in Fig. 4b. After 15 h, the UVB group had the widest scratch distance, whereas the Ctrl and hUC-MSC-CM groups had similar shorter scratch distances. However, after 30 h, the scratches in the hUC-MSC-CM group were closed, more so than those in the Ctrl group, whereas the scratches in the UVB group were not yet closed. These results indicated that UVB-irradiated HaCaT cells had slower motility than normal HaCaT cells. However, treatment with hUC-MSC-CM restored and even improved the mobility and migration ability of HaCaT cells. Together, the results indicated that hUC-MSC-CM promoted the recovery of cell motility in the cellular photoaging model.

### Effect of hUC-MSC-CM on key genes and proteins in UVB-irradiated HaCaT cells

**Fig. 5:**
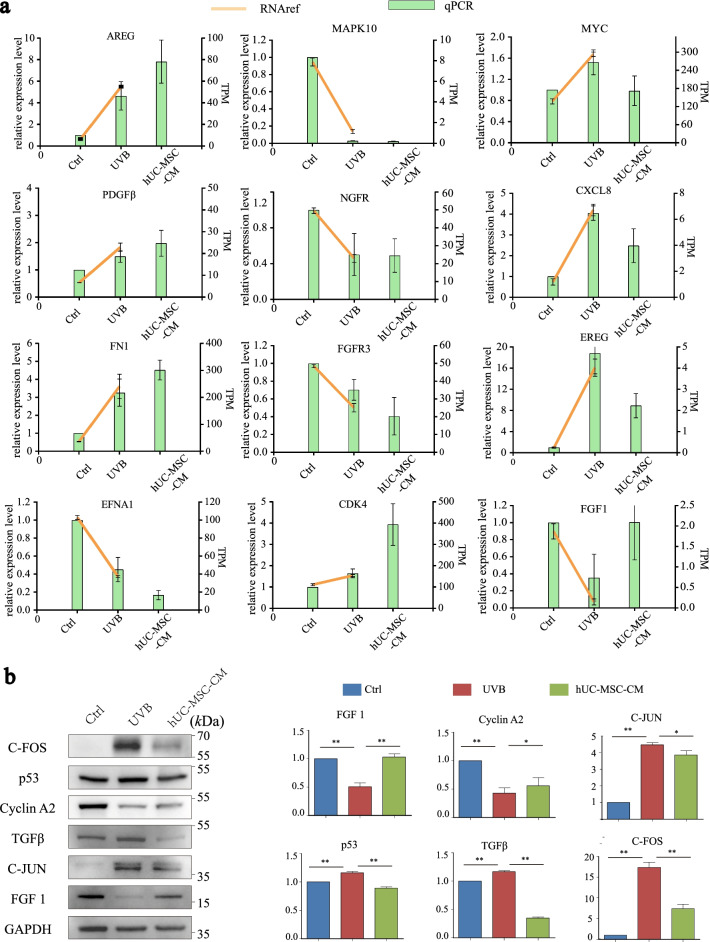
Effects of hUC-MSC-CM on ageing-related genes and proteins in UVB-irradiated HaCaT cells. **a** qRT-PCR analysis to verify the expression of key genes. The abscissa represents the group, the green bar graph represents the relative gene expression of the qRT-PCR results (left *Y*-axis), and the yellow line represents the transcript per million values of the genes in the transcriptome (right *Y*-axis). **b** Protective effects of hUC-MSC-CM on ageing-related proteins. Ctrl group (UVB-, CGM), UVB group (UVB+, hUC-MSC-CM), and hUC-MSC-CM group (UVB+, hUC-MSC-CM). The abscissa represents the group, and the ordinate represents the relative protein expression level. Statistical results are expressed as the mean ± standard deviation. **, *p* < 0.01; *, *p* < 0.05, *n* = 3

To verify the reliability of the transcriptome data, some key genes (Fig. 1b) were selected for qRT-PCR analysis based on the protein–protein interaction network (Additional file 3: Fig. S2). The expression of key genes based on qRT-PCR analysis was consistent with the omics results. Among them, the expression of MYC, CXCL8, FGF-1, and EREG opposed the original trend after intervention with hUC-MSC-CM (Fig. 5a), suggesting these key genes may be responsible for the anti-photoaging effects of hUC-MSC-CM on UVB-irradiated HaCaT cells.

To further explore the protective effects of hUC-MSC-CM on UVB irradiation damage, the expression of several common ageing-related proteins was verified using western blotting (Fig. 5b). The results showed that UVB irradiation induced a significant increase in the expression of C-FOS (17.38-fold), C-JUN (4.47-fold), p53 (1.16-fold), and TGFβ (1.16-fold), whereas hUC-MSC-CM significantly inhibited UVB-induced overexpression of C-FOS (7.43-fold), C-JUN (3.86-fold), p53 (0.88-fold), and TGFβ (0.34-fold). In addition, UVB irradiation caused a decrease in cyclin A2 protein expression (0.32-fold) and FGF1 (0.51-fold), whereas hUC-MSC-CM treatment increased the protein expression of cyclin A2 (0.62-fold) and FGF1 (1.02-fold).

## Discussion

The average daily UVB exposure of high school students in Japan is reported as 8.01 mJ/cm^2^/day [46]. In the current study, HaCaT cells were exposed to 20 mJ/cm^2^ UVB irradiation after starvation for 24 h, which is equal to nearly three days of UVB irradiation. Photoaging-related studies commonly use in vitro models containing 10% FBS in the culture medium. Various growth factors contained in the FBS can inhibit the biological effects caused by UVB irradiation [47]. Compared to the traditional serum-containing photoaging model, the serum-free model does not contain anti-photoaging components such as growth factors, and the photoaging phenotype will be accurate and significant. The serum-free model is also helpful for the subsequent search for genes and proteins that play a key role in the anti-photoaging effect of hUC-MSC-CM. To the best of our knowledge, this is the first study to explore the relationship between photoaging and SASP using a cellular model comprising serum-starved HaCaT cells combined with multi-omics correlation analysis. In addition, the reliability of the HaCaT cell photoaging model was further validated by comparing the cell proliferation, apoptosis, cell cycle, ROS production, and cell motility between the UVB and Ctrl groups.

Transcriptomics analysis suggested that UVB-affected pathways were mainly those involved in cell proliferation, differentiation, and apoptosis [48–50], including ribosome, microRNAs in cancer, cell cycle, and cellular senescence, protein processing in the endoplasmic reticulum, and p53 signalling pathways. Exposure to oxidative stress or UVB irradiation causes endoplasmic reticulum stress, which initiates cell death [48]. Activation of p53 can lead to cell cycle arrest, cell senescence, apoptosis and inhibit tumour development [51], among which cell cycle arrest and apoptosis are two major protective responses against UVB-induced cellular damage and mutagenesis [52]. Later experiments further confirmed that UVB irradiation led to increased cell apoptosis and cell cycle arrest. The expression of selected genes involved in the SASP process from the transcriptome was verified by qRT-PCR, revealing that the expression of all key genes differed significantly before and after UVB treatment, and most trends were consistent. These results confirmed that the HaCaT cell photoaging model was successfully constructed.

Protein expression lags behind mRNA expression. Correlation analysis between transcriptomics and proteomics data revealed that the expression trends of many mRNAs and proteins were inconsistent. However, pathways closely related to cell senescence, proliferation, and apoptosis were enriched in both sets of data, suggesting UVB-related effects on cellular senescence and the cell cycle. Furthermore, differential proteins were more enriched in cellular senescence pathways. In addition, differential proteins were enriched in the focal adhesion pathway. Focal adhesion refers to the contact point between the cell and the ECM, which plays an important role in regulating tissue remodelling and cell migration [53]. These results were also confirmed by cell migration experiments, in which UVB irradiation reduced cell motility.

Stem cell-conditioned medium and exosomes have been widely used in skin anti-ageing research. Choi et al. applied adipose-derived stem cell (ASC) exosomes to a UVB-induced fibroblast photoaging model, reporting that ASC exosomes could enhance the proliferation, migration, and invasion of aged HDF cells [54]. Furthermore, a previous study demonstrated that the antioxidant capacity of the ASC-conditioned medium was 1.8 times higher than ordinary complete medium [55]. The results of the current study indicated that hUC-MSC-CM inhibited UVB-induced HaCaT cell apoptosis, ROS production, and promoted cell proliferation and migration. Apoptosis is affected by multiple signalling pathways, including the p53 signalling pathway [56]. p53 was detected in both transcriptomics data and western blot experiments, revealing that p53 expression was increased after UVB irradiation and decreased after the addition of hUC-MSC-CM. These results indicated that hUC-MSC-CM could inhibit apoptosis by reducing the expression of p53. UVB irradiation can generate large amounts of ROS, which is also a major factor in skin ageing [57]. A large amount of ROS affects the mitochondrial membrane potential and therefore the energy production by mitochondria, which is needed for cell migration [58]. Therefore, cell motility also decreased after UVB irradiation. The experimental results indicated that hUC-MSC-CM can effectively reduce the production of intracellular ROS and promote the recovery of cell motility. Cell cycle arrest can lead to a decrease in the ability of cells to proliferate [59]. UVB irradiation blocked the transition from the G1 phase to the G2 phase of the cell cycle in HaCaT cells, resulting in decreased cell proliferation. Furthermore, hUC-MSC-CM promoted recovery of cell proliferation ability by inhibiting G1 phase arrest.

The qRT-PCR results indicated that the MYC, CXCL8, FGF-1, and EREG genes play an important role in the anti-photoaging activity of hUC-MSC-CM, although the underlying mechanisms require further research. MYC is a proto-oncogene involved in the regulation of various cellular functions, including cell proliferation [60]. CXCL8 is an important inflammatory factor and characteristic molecule of SASP. FGF1 is also known as an acidic fibroblast growth factor. A previous study added purified FGF1 to the culture medium of HaCaT cells irradiated by UVB, reporting that FGF1 can effectively improve the survival rate of HaCaT cells by 89% [61]. EREG has previously been identified as a potential target for treating skin damage caused by UVB [62].

The results of the western blot indicated that C-FOS, C-JUN, cyclin A2, TGFβ, p53, and FGF1 are key proteins involved in the anti-photoaging effects of hUC-MSC-CM. C-FOS and C-JUN are key molecules in the MAPK pathway and are involved in cell apoptosis. UVB irradiation has been shown to activate the MAPK signalling pathway, resulting in transcription factors C-FOS and C-JUN forming heterodimeric AP-1 [63]. AP-1 is a key molecule involved in the inhibition of UV-induced apoptosis of stem cells [64]. Cyclin A2 is a key protein in cell cycle regulation and can participate in the regulation of the G1 phase of the cell cycle with CDK [65]. TGFβ can regulate the cell cycle by first blocking the translation of CDK4 and then inhibiting CDK2-cyclin E, resulting in cell cycle arrest in the G1 phase [66]. Therefore, cyclin A2 and TGFβ may be key targets of hUC-MSC-CM by inhibiting UVB-induced G1 phase arrest of HaCaT cells. FGF1 has been mentioned and validated in previous qRT-PCR studies and induces cell proliferation.

Moreover, the results of the current study indicated that the anti-photoaging effects of hUC-MSC-CM may be related to the state of hUC-MSCs. The logarithmic growth phase is used to harvest hUC-MSCs, and the anti-photoaging ability of hUC-MSCs-CM is stronger near the first cell passage. This may be related to the quality of exosomes in hUC-MSC-CM. The composition of the stem cell-conditioned medium is complex, including a variety of growth factors, peptides, miRNAs, exosomes, etc. Although various studies have demonstrated the anti-photoaging effects of these factors, we believe exosomes are likely to be the key anti-photoaging factor. Studies have shown that the anti-photoaging ability of hUC-MSC-CM is greatly weakened if exosomes are removed [64]. Exosomes, which are small extracellular vesicles secreted by cells, are rich in proteins, lipids, and nucleic acids and can transmit signals [67]. We speculate that exosomes secreted by hUC-MSCs in the logarithmic growth phase contain components that are more conducive to cell proliferation, movement, and invasion. Moreover, many studies have shown that the ability of stem cells to resist photoaging is enhanced when cells are pre-treated with certain external conditions [1], including direct pretreatment with stimuli (such as inflammatory factors), gene manipulation, and variations in culture conditions, such as three-dimensional (3D) and hypoxic culture [68]. Preconditioning ASCs with specific biochemical factors is an important approach to improving cell function and therapeutic potential. In a mouse glioblastoma model, Li et al. reported that pretreatment of ASCs with TGFβ upregulated the expression of CXCR4 and significantly improved the anticancer effect [69]. Han et al. found that the overexpression of SOX2 and OCT4 in ASCs promoted their proliferation and differentiation potential, reverting to a more primitive state [70]. Compared to 2D culture, the multicellular structure achieved in 3D culture has superior biological simulation advantages [71]. 3D culture has been shown to increase ECM production, enhance cell proliferation, and increase levels of anti-inflammatory and pro-angiogenic cytokines [68]. Furthermore, Rhijn et al. found that ASCs cultured under 1% O_2_ could inhibit mitogen-stimulated CD4 and CD8 T lymphocyte proliferation, thus enhancing the immunomodulatory effect of ASCs [38]. Furthermore, hypoxia-pre-treated ASC-conditioned medium promoted improved endothelial cell survival and tube formation [72]. Taken together, these studies suggest that a variety of methods may enhance the strength of the anti-photoaging ability of hUC-MSCs-CM, thus enhancing its therapeutic function.

## Conclusion

Multi-omics correlation analysis indicated that serum starvation of HaCaT cells followed by irradiation with 20 mJ/cm^2^ UVB in vitro successfully established a novel cellular photoaging model. Furthermore, treatment with hUC-MSC-CM was shown to inhibit cell apoptosis and cell cycle arrest caused by UVB, reduce ROS production, promote cell proliferation, and enhance cell motility. Taken together, the study findings indicate that hUC-MSC-CM holds promise as an effective anti-photoaging agent that can be used against skin ageing.

## Supplementary Information

**Additional file 1: Table S1.** Primer information for qRT-PCR.

**Additional file 2: Fig. S1.** SASP transcriptional regulation process.

**Additional file 3: Fig. S2.** Protein–protein interaction diagram.

## Data Availability

The raw data for the transcriptomics and proteomics analyses have been deposited in the SRA of NCBI (https://submit.ncbi.nlm.nih.gov/subs/sra/). Other datasets generated during and/or analysed in the current study are available from the corresponding authors upon reasonable request.

## Abbreviations

ASC: Adipose-derived stem cell
BP: Biological process
CC: Cell component
Ctrl: Control
CAT: Catalase
ECM: Extracellular matrix
FBS: Foetal bovine serum
FDR: False discovery rate
GCSF: Granulocyte colony-stimulating factor
GO: Gene Ontology
HDF: Human dermal fibroblast
GPX: Glutathione peroxidase
CGM: Complete growth medium
hUC-MSC-CM: Human umbilical cord mesenchymal stem cell-conditional medium
IL: Interleukin
KEGG: Kyoto Encyclopedia of Genes and Genomes
MF: Molecular function
MMP: Matrix metalloproteinases
PBS: Phosphate-buffered saline
ROS: Reactive oxygen species
SASP: Senescence-associated secretory phenotype
TNF-α: Tumour necrosis factor-α
UVB: Ultraviolet B rays
UC-MSC: Umbilical cord mesenchymal stem cell
UC-MSC SFM: UC-MSC serum-free medium
