# Supplementary material for: Multi-omics analysis of an in vitro photoaging model and protective effect of umbilical cord mesenchymal stem cell-conditioned medium
Source: Stem Cell Res Ther. 2022 Sep 2;13:435. doi: 10.1186/s13287-022-03137-y (PMC9438153; doi:10.1186/s13287-022-03137-y)
Supplement: Supplementary file 1 — Additional file 1: Table S1. Primer information for qRT-PCR. [file 13287_2022_3137_MOESM1_ESM.docx]

Supplementary Table 1. Primer information for qRT-PCR.

| Primer name | | Primers sequence (5’→3’) |
| --- | --- | --- |
| CXCL8 |  | F：ACTGAGAGTGATTGAGAGTGGAC |
|  |  | R：AACCCTCTGCACCCAGTTTTC |
| FN1 |  | F：GTTCGGGAGGAGGTTGTTACC |
|  |  | R：GAGTCATCTGTAGGCTGGTTTAGG |
| FGFR3 |  | F：GCATCCTCACTGTGACATCAAC |
|  |  | R：CCTGGCGAGTACTGCTCAAA |
| EREG |  | F：TTATGGGAGGCTCCTTCATC |
|  |  | R：GCCTTCGTTTACCCTAGCAC |
| CDK4 |  | F：TTTTGAGCATCCCAATGTTGTC |
|  |  | R：TCGACGAAACATCTCTTGATCT |
| FGF-1 |  | F：CAATGTTTGGGCTAAGACCTG |
|  |  | R：GGCTGTGAAGGTGGTGATTT |
| PDGFβ |  | F：GGCTGTGACTTAGACAGGCT |
|  |  | R：TTTGAGTGAGACAGGCACCC |
| MAPK10 |  | F：CTTCCCAGATTCCCTCTTCC |
|  |  | R：GCTGGGTCATACCAGACGTT |
| CCNG2 |  | F：AAGAAGAGAGATTCCAACC |
|  |  | R：CCAGCAAAAAAGAACAGAC |
| EFNA1 |  | F：AGGTGCGGGTTCTACATAGCA |
|  |  | R：AGTCCAGGCAAGTGGGAAGA |
| AREG |  | F：GATACTCGGCTCAGGCCATTAT |
|  |  | R：CAAATCCATCAGCACTGTGGTC |
| NGFR |  | F：CCATCTTGGCTGCTGTGGTC |
|  |  | R：CCACTGTCGCTGTGCAGTTTC |
| GAPDH |  | F：CAGGAGGCATTGCTGATGAT |
|  |  | R：GAAGGCTGGGGCTCATTT |

This table is the list of primers mentioned in Materials and Methods (qRT-PCR). The name of the primer is listed on the left, and the specific sequence of the primer is on the right.
