# Supplementary material for: Multi-omics analysis of an in vitro photoaging model and protective effect of umbilical cord mesenchymal stem cell-conditioned medium
Source: Stem Cell Res Ther. 2022 Sep 2;13:435. doi: 10.1186/s13287-022-03137-y (PMC9438153; doi:10.1186/s13287-022-03137-y)
Supplement: Supplementary file 2 — Additional file 2: Fig. S1. SASP transcriptional regulation process. [file 13287_2022_3137_MOESM2_ESM.docx]

Supplementary Figure 1. SASP transcriptional regulation process.


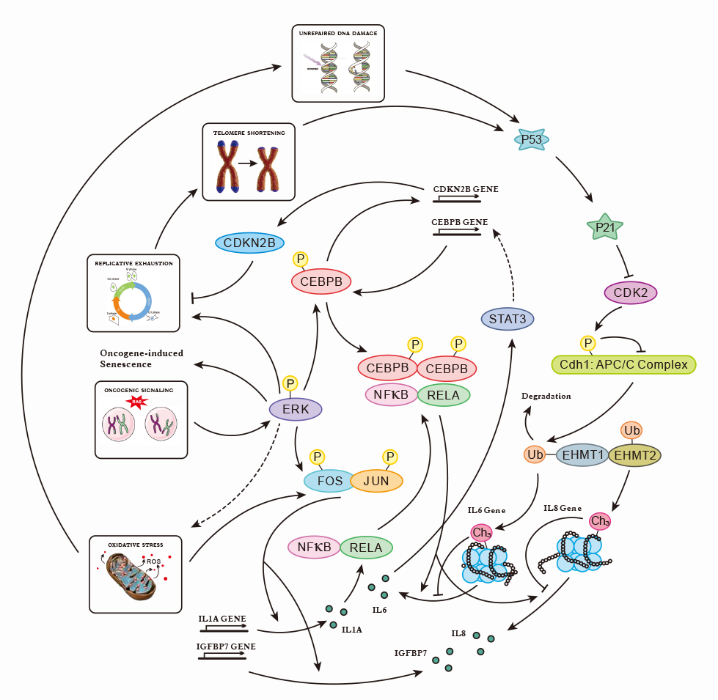


This picture is the SASP transcriptional regulation process mentioned in results (Transcriptomics results ). Most of the genes involved in SASP will be picked out in the transcriptome.
