# Supplementary material for: Multi-omics analysis of an in vitro photoaging model and protective effect of umbilical cord mesenchymal stem cell-conditioned medium
Source: Stem Cell Res Ther. 2022 Sep 2;13:435. doi: 10.1186/s13287-022-03137-y (PMC9438153; doi:10.1186/s13287-022-03137-y)
Supplement: Supplementary file 3 — Additional file 3: Fig. S2. Protein–protein interaction diagram. [file 13287_2022_3137_MOESM3_ESM.docx]

Supplementary Figure 2. Protein-protein interaction diagram.
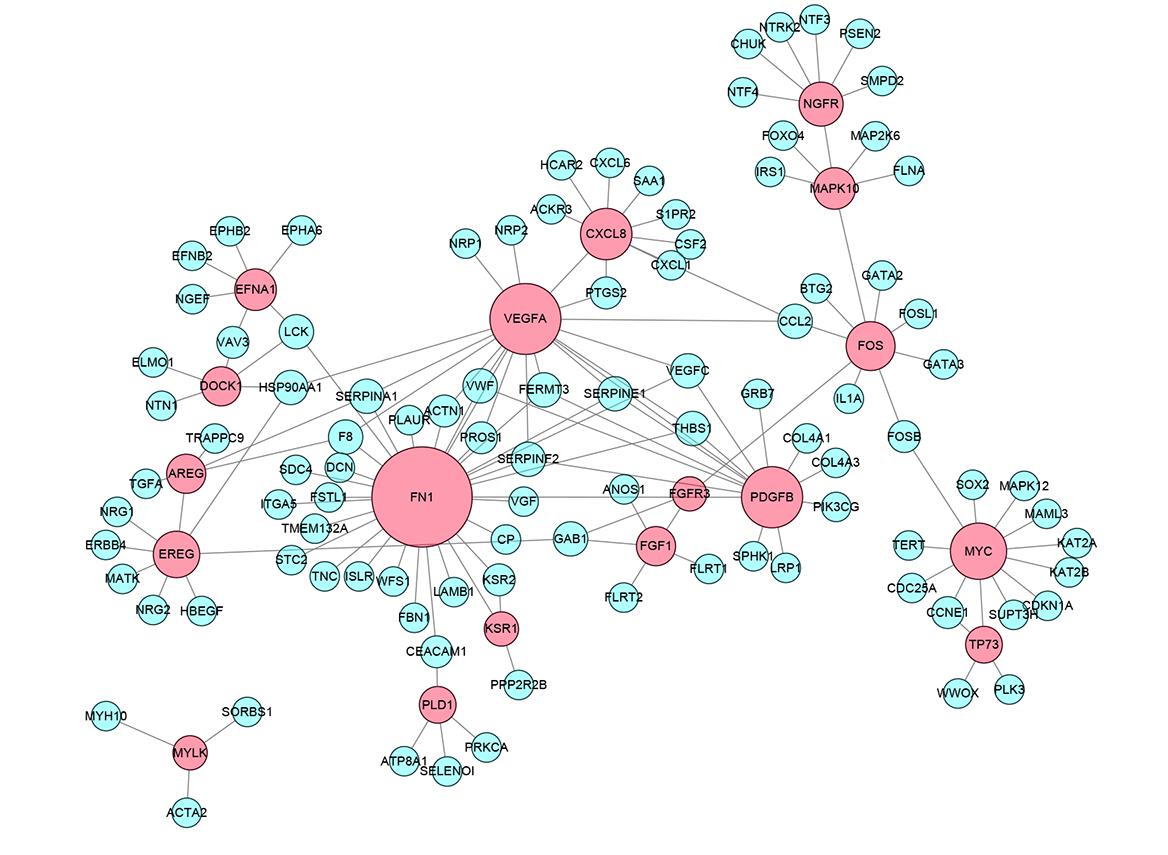


This picture is the protein-protein interaction diagram mentioned in results (Effect of hUC-MSC-CM on key genes and proteins in UVB-irradiated HaCaT cells). This picture uses the Dr.Tom system of China BGI to determine the key genes of qRT-PCR based on the protein-protein interaction relationship. The key genes had been marked in red.
